# Acromioclavicular Ganglion Cyst: A Rare Case Report

**DOI:** 10.7759/cureus.54089

**Published:** 2024-02-12

**Authors:** Tushar Chaudhari, Archit Gupta

**Affiliations:** 1 Orthopaedics, Dr. D. Y. Patil Medical College, Hospital & Research Centre, Pune, IND

**Keywords:** ac joint, surgical management, shoulder pain, ganglion cyst, acromioclavicular cyst

## Abstract

Acromioclavicular ganglion cysts are uncommon, with only a limited number of cases reported in the medical literature. This case report presents a unique instance of an acromioclavicular ganglion cyst in an 81-year-old male patient, outlining the clinical presentation, diagnostic approach, and successful surgical management. The purpose of this report is to contribute to the existing body of knowledge on this rare condition and highlight the importance of accurate diagnosis and appropriate intervention.

## Introduction

Ganglion cysts are fluid-filled lesions commonly arising from joints or tendon sheaths, with the acromioclavicular joint being an infrequent site of occurrence. A ganglion cyst usually arises as sequelae of chronic AC joint arthritis or repeated microtrauma [1]. The rarity of acromioclavicular ganglion cysts poses diagnostic challenges, often leading to delayed treatment and prolonged patient discomfort. The case report's purpose is to raise awareness of this uncommon ailment by highlighting the need for early diagnosis and suitable treatment [2].

## Case presentation

**Figure 1:**
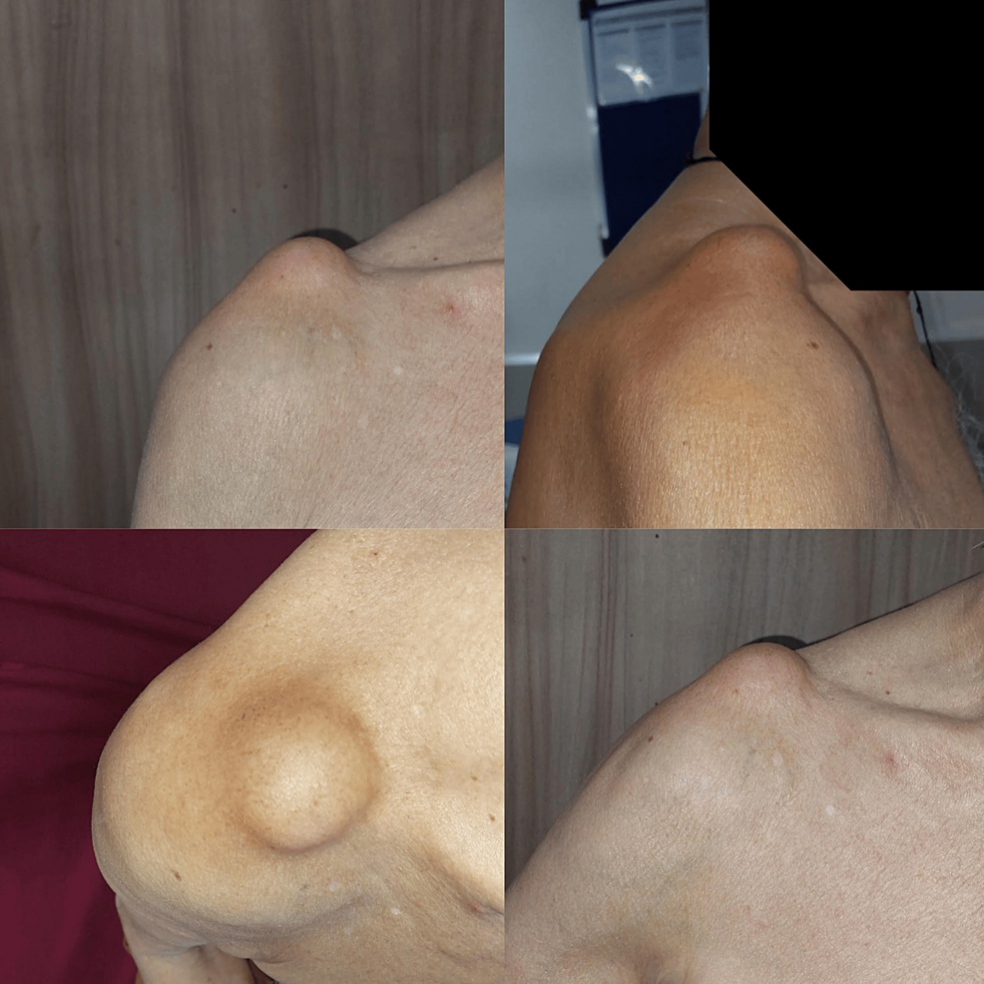
Clinical images showing the location of the swelling over the patient's shoulder

A male in his 80s presented to the orthopaedic clinic with a chief complaint of persistent right shoulder pain for the past three months and aggravated for the past 15 days. The pain was localized over the acromioclavicular joint and exacerbated by overhead activities. The patient reported a history of trauma but noted a gradually worsening discomfort over the past three months. Clinical examination revealed a palpable swelling measuring 4x4 cm, over the acromioclavicular joint, tender to touch, with no signs of erythema or warmth (Figure 1). The range of motion was restricted as the patient could not do overhead abduction. The swelling did not subside with overhead abduction. Neurovascular examination of the upper extremity was unremarkable.

Diagnostic workup

**Figure 2:**
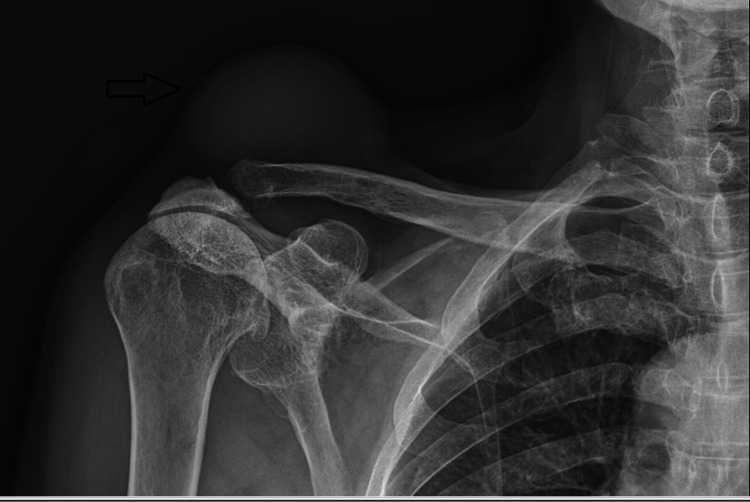
Plain radiograph of the right shoulder showing a soft tissue shadow (black arrow)

**Figure 3:**
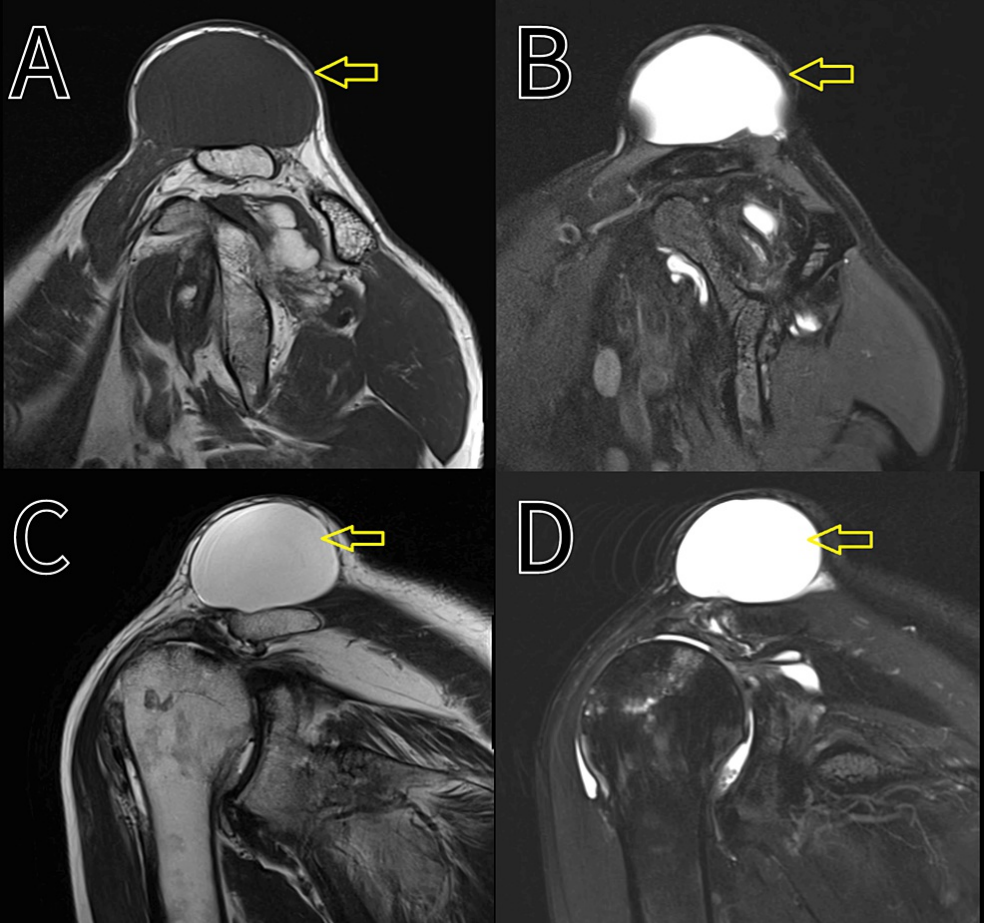
MRI showing a localised soft tissue swelling A: T1-weighted image, B: T2-weighted image, C: T2-weighted image, D: proton density fat-saturated (PDfs) image

Initial imaging studies, including plain radiographs, revealed no abnormalities in the bony structures of the acromioclavicular joint (Figure 2). MRI was subsequently performed, demonstrating a well-defined cystic lesion adjacent to the joint capsule, consistent with a ganglion cyst (Figure 3). The cyst exhibited no communication with the joint space.

Treatment

**Figure 4:**
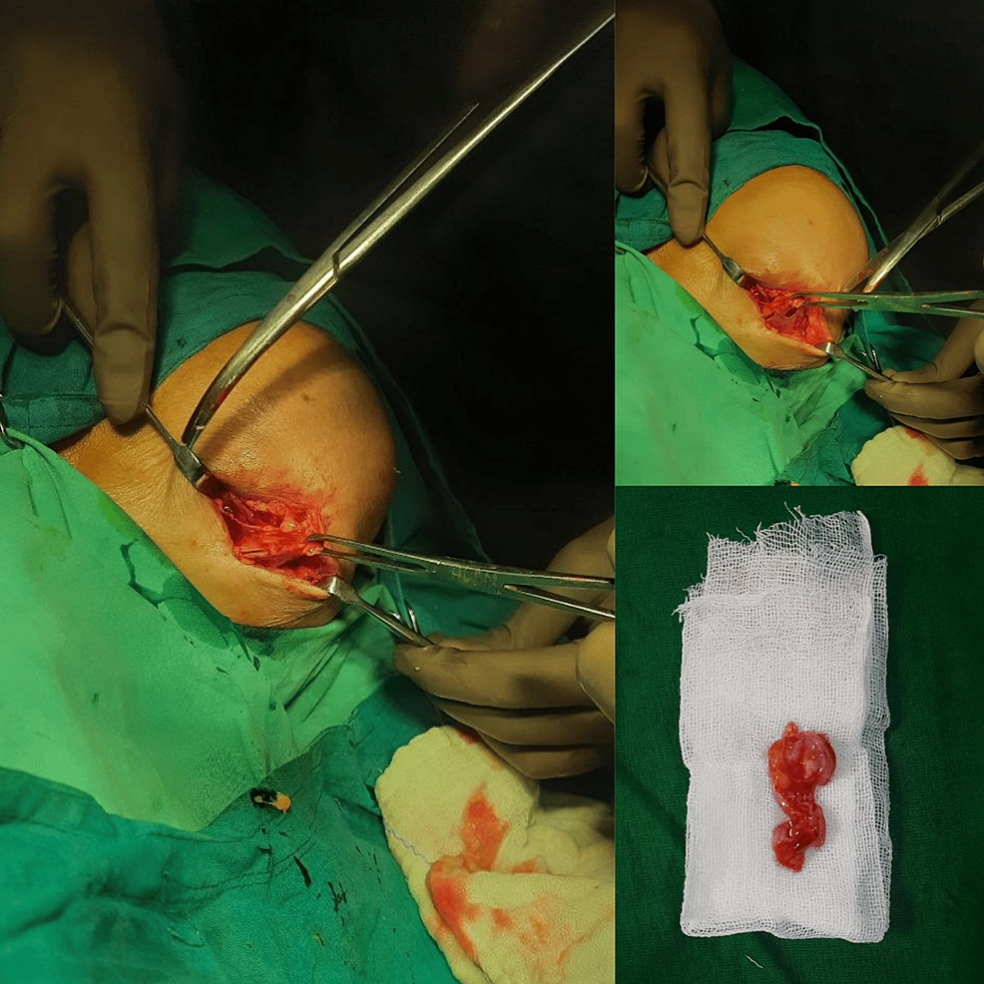
Intraoperative images showing the steps during the excision of the cyst The excised cyst wall is visible on the bottom right.

Surgical intervention was recommended given the persistent symptoms and the impact on the patient's daily activities. The patient was positioned in a reclined Fowler position. The cyst has been excised en bloc using an open technique and a Langerhans line incision while under local anesthesia. It was discovered to have a mucinous material (Figure 4). Intraoperative findings confirmed the presence of a cystic mass originating from the acromioclavicular joint capsule. Histopathological examination was suggestive of a benign cystic lesion most probably a ganglion cyst. The excised cystic tissue had a thick fibrocollagenous wall with a markedly attenuated lining and focal myxoid change. The lumen contained a focal eosinophilic material. The analysis revealed no significant inflammation or malignancy.

Outcome

Postoperatively, the patient experienced significant shoulder pain relief and improved range of motion. Follow-up examinations at three and six months revealed no recurrence of symptoms, and the patient reported a return to normal daily activities without restrictions.

## Discussion

Cysts in the acromioclavicular joint are uncommon outcomes of advanced arthritis in the AC joint and persistent rotator cuff tears. They have been categorized into type 1 and type 2 cysts based on their origins. Type 1 cysts manifest in advanced AC joint arthritis, involving synovial inflammation, and these cysts have been confined to the joint without any association with a rotator cuff tear. On the other hand, the shape of the rotator cuff is associated with the pathophysiology of type 2 cysts. The humeral head’s superior migration is a danger related to a full rotator cuff tear, particularly the supraspinatus tear in cuff tear arthropathy. This migration can lead to irritation and degradation of the AC joint capsule.

A ganglion cyst is created when an increased production of synovial fluid escapes into the AC joint capsule due to the development of a check valve. As a result, the fluid builds up in the subcutaneous layer, where the cyst forms. Acromioclavicular ganglion cysts are rare entities, and their clinical presentation can mimic other shoulder pathologies. Imaging studies, particularly MRI, play a crucial role in accurate diagnosis.

Surgical excision, as performed in this case, can provide effective and lasting relief from symptoms. Because aspiration-related recurrence is linked to AC joint cysts, current treatment recommendations mandate surgical intervention [3]. Kontakis et al. used subacromial bursectomy and distal clavicular resection to treat an isolated AC joint cyst, an arthritis undamaged rotator cuff, and an AC joint [4]. In their findings, Rohit et al. indicated that conservative therapy might completely resolve the AC joint cyst [2]. The different alternatives include humeral head replacement, distal clavicular resection, complete shoulder arthroplasty, arthroscopic debridement with rotator cuff tear repair, and shoulder arthrodesis [1]. In order to improve the excision location, Skedros et al. [5] relocated the anterior deltoid and used an allograft patch to cover the surfaces of the removed bones and the remaining AC joint ligaments. Because of the check valve, which keeps the fluid from passing through it, conservative therapy is never recommended.

This report underscores the importance of considering acromioclavicular ganglion cysts in the differential diagnosis of shoulder pain and highlights the successful outcome achievable through timely surgical intervention.

## Conclusions

This case report sheds light on the diagnostic and therapeutic challenges posed by acromioclavicular ganglion cysts, emphasizing the significance of a comprehensive clinical assessment and advanced imaging techniques. The rarity of this condition underscores the need for heightened awareness among healthcare professionals to ensure timely and accurate diagnosis.

The successful surgical management of the presented case highlights the efficacy of excision in alleviating symptoms and restoring functional capacity. While conservative measures may be considered in certain instances, surgical intervention proves to be a viable and definitive solution for cases marked by persistent pain and limited range of motion. Ultimately, this report serves as a reminder to clinicians that while acromioclavicular ganglion cysts may be infrequent, they should be considered in the differential diagnosis of shoulder pain, especially when conventional treatments prove ineffective.

Timely recognition and appropriate management can significantly improve patient's quality of life, emphasizing the importance of a multidisciplinary approach in addressing uncommon musculoskeletal pathologies. Continued research and reporting of similar cases will contribute to the collective knowledge base, ultimately benefiting patient care and outcomes.
